# Joint small-angle X-ray and neutron scattering data analysis of asymmetric lipid vesicles

**DOI:** 10.1107/S1600576717000656

**Published:** 2017-02-28

**Authors:** Barbara Eicher, Frederick A. Heberle, Drew Marquardt, Gerald N. Rechberger, John Katsaras, Georg Pabst

**Affiliations:** a Instiute of Molecular Biosciences, Biophysics Division, University of Graz, Austria; b BioTechMed-Graz, Graz, 8010, Austria; c The Bredesen Center for Interdisciplinary Research and Graduate Education, University of Tennessee, Knoxville, TN, USA; d Joint Institute for Biological Sciences, Oak Ridge National Laboratory, Oak Ridge, TN, USA; e Biology and Soft Matter Division, Oak Ridge National Laboratory, Oak Ridge, TN, USA; f Instiute of Molecular Biosciences, University of Graz, Austria; g Omics-Center Graz, BioTechMed-Graz, Austria; h Shull Wollan Center, Oak Ridge National Laboratory, Oak Ridge, TN, USA

**Keywords:** lipid bilayers, asymmetric membranes, transbilayer coupling, small-angle X-ray scattering (SAXS), small-angle neutron scattering (SANS), joint SAXS/SANS analysis, scattering density profile models

## Abstract

Low- and high-resolution models describing the internal transbilayer structure of asymmetric lipid vesicles have been developed. These models can be used for the joint analysis of small-angle neutron and X-ray scattering data. The models describe the underlying scattering length density/electron density profiles either in terms of slabs or through the so-called scattering density profile, previously applied to symmetric lipid vesicles. Both models yield structural details of asymmetric membranes, such as the individual area per lipid, and the hydrocarbon thickness of the inner and outer bilayer leaflets. The scattering density profile model, however, comes at a cost of increased computational effort but results in greater structural resolution, showing a slightly lower packing of lipids in the outer bilayer leaflet of ∼120 nm diameter palmitoyl­oleoyl phosphatidyl­choline (POPC) vesicles, compared to the inner leaflet. Analysis of asymmetric dipalmitoyl phosphatidylcholine/POPC vesicles did not reveal evidence of transbilayer coupling between the inner and outer leaflets at 323 K, *i.e.* above the melting transition temperature of the two lipids.

## Abstract 2

Low- and high-resolution scattering length density models for the joint analysis of small-angle neutron and X-ray scattering experiments on asymmetric lipid vesicles are presented.

## 1. Abbreviations   


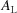
: area per lipid

aLUV: asymmetric large unilamellar vesicle

aSDP: asymmetric scattering density profile

CD: cyclodextrin

CG: glycerol group

DE: differential evolution

DPPC: dipalmitoyl phophatidylcholine


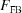
: form factor of a flat bilayer sheet

GC: gas chromatography

HC: hydrocarbon group

LUV: large unilamellar vesicle


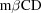
: methyl-β-cyclodextrin

M: methyl group

MLV: multilamellar vesicles

MS: mass spectrometry

PC: phophatidylcholine

POPC: palmitoyl-oleoyl phosphatidylcholine

SDD: sample–detector distance

SDP: scattering density profile

SFF: separated form factor

SLD: scattering length density

UPLC: ultra-performance liquid chromatography


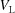
: lipid molecular volume

## 2. Introduction   

Most biological membranes are asymmetric. For example, mammalian plasma membranes contain mainly phosphatidyl­choline (PC) and sphingomyelin in their outer (exoplasmic) leaflets, while phosphatidylserine and phosphatidylethanol­amine are the major lipid groups of their inner (cytosolic) leaflets (Verkleij *et al.*, 1973 ▸; Devaux, 1991 ▸). Bilayer asymmetry is thought to affect various membrane properties including electrostatic potential, surface charge, permeability and stability, in addition to structural parameters such as bilayer thickness, and even the thicknesses of the individual leaflets (Devaux, 1991 ▸). However, until recently progress in studying the biophysics of asymmetric bilayers has been hampered by the lack of protocols pertaining to their formation (Marquardt, Geier & Pabst, 2015 ▸).

In a series of papers, London and co-workers introduced a method using cylcodextrin (CD)-mediated lipid exchange for producing solvent-free free-floating asymmetric vesicles of different sizes (Cheng *et al.*, 2009 ▸; Chiantia & London, 2013 ▸). (Note that the name asymmetric lipid vesicle refers to a vesicle whose bilayer leaflets are compositionally different.) We recently modified this technique to produce stress-free asymmetric large unilamellar vesicles (aLUVs) amenable to interrogation by different biophysical techniques (Heberle *et al.*, 2016 ▸). These include small-angle X-ray and neutron scattering (SAXS and SANS, respectively), techniques which are well known for their abilities to probe membrane structure at the sub-nanometre scale without the need for extrinsic probes (Pabst *et al.*, 2010 ▸; Marquardt, Heberle *et al.*, 2015 ▸). Over the years, several concepts have been developed to model symmetric lipid bilayers in terms of scattering length density (SLD) profiles. In general, these models consist of step functions or ‘slabs’ (*e.g.* Riske *et al.*, 2001 ▸; King *et al.*, 1985 ▸; Pencer & Hallett, 2000 ▸; Schmiedel *et al.*, 2001 ▸), Gaussians (*e.g.* Wiener & White, 1992 ▸; Pabst *et al.*, 2000 ▸; Nagle & Tristram-Nagle, 2000 ▸), or some combination of the two. A particularly influential method of determining membrane structure at high resolution is the scattering density profile (SDP) model developed by Kučerka and co-workers (Klauda *et al.*, 2006 ▸; Kučerka *et al.*, 2008 ▸), which allows for the joint analysis of X-ray and neutron data. More recently, an all-atom model for the SLD was developed, which has an even higher internal resolution than the SDP (Fogarty *et al.*, 2015 ▸).

Brzustowicz & Brunger (2005 ▸) were the first to report a smooth SLD model function to analyze SAXS data from asymmetric (*i.e.* noncentrosymmetric) lipid vesicles. Later, Kučerka and co-workers described an SDP-based model for asymmetric bilayers (Kučerka, Pencer, Sachs *et al.*, 2007 ▸), which exploited SANS/SAXS contrast variation (Pabst *et al.*, 2010 ▸; Marquardt, Heberle *et al.*, 2015 ▸). However, their model did not consider isotopic labeling of only one bilayer leaflet (Heberle *et al.*, 2016 ▸), which is needed to precisely define the center of the asymmetric bilayer in order to disentangle leaflet-specific thicknesses and lipid packing densities. To this end, we have developed an asymmetric SDP model (‘aSDP’) that allows for this feature. In addition, we describe a slab model that also allows for the joint analysis of SAXS and SANS data, but at a lower spatial resolution. The main advantage of the slab model is that fewer parameters are needed to fit the data.

Here, we evaulate the efficacy of both the slab and SDP models using isotopically labeled aLUVs composed of palmitoyloleoyl phosphatidylcholine (POPC) and dipalmitoyl phosphatidylcholine (DPPC), as well as their deuterated variants. Despite the significant difference in spatial resolution, the two models yield comparable values for the area per lipid 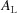 and the thicknesses of the inner and outer hydrocarbon layers 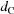. However, the quality of the fits, as judged by their reduced 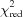 values, are better when using the aSDP model. Finally, our analysis of fluid DPPC/POPC aLUVs revealed that the inner and outer membrane leaflets are structurally decoupled from each other at 323 K, above the melting transition temperature of the two lipids.

## 3. Materials and methods   

### 3.1. Sample preparation   

All lipids, including their isotopes (POPC-d13, POPC-d31, POPC-d44, DPPC-d13, DPPC-62) were purchased from Avanti Polar Lipids (Alabaster, AL, USA) and used without further purification (see Fig. S6 of the supporting information for details of chemical structures). D
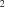
O (99.8%) was obtained from Alfa Aesar (Ward Hill, MA, USA) and from Euroiso-top (Saarbrücken, Germany). Methyl-β-cyclocextrin (mβCD) was obtained from Sigma–Aldrich (St Louis, MO, USA). All solvents were of pro analysis quality. Lipid stock solutions were prepared by dissolving weighed amounts of dry lipid powder in chloroform. The lipid stock solution concentration was determined to within 1% by inorganic phosphate assay (Kingsley & Feigenson, 1979 ▸). Appropriate volumes of the stock solutions were dried under a stream of nitrogen and placed under vacuum for at least 12 h, leading to a thin lipid film on the bottom of a glass vial.

aLUVs were prepared using cyclodextrin-mediated lipid exchange as previously described (Heberle *et al.*, 2016 ▸). Briefly, acceptor vesicles composed of the inner leaflet lipids were prepared by first hydrating the dry lipid films in a 20 m*M* NaCl aqueous solution made from 18 MΩ cm water (lipid concentration 10 mg ml^−1^). The resulting multilamellar vesicles (MLVs) were incubated at 313 K for 1 h with intermittent vortex mixing, followed by five freeze/thaw cycles using liquid nitrogen. LUVs were prepared using a hand-held mini-extruder (Avanti Polar Lipids, Alabaster, AL, USA) with a 100 nm pore-diameter polycarbonate filter. The MLV suspension was passed through the filter a total of 31 times at room temperature. LUV formation was facilitated by doping the lipids with 5 mol% POPG or POPG-d31 (matching the isotopic composition of the inner leaflet POPC or POPC-d31 lipids). Such low amounts of the charged lipid were previously shown to not affect the membrane structure of phosphatidylcholines (Kučerka, Pencer, Sachs *et al.*, 2007 ▸). Vesicle size was measured by dynamic light scattering using a Zetasizer NANO ZSP (Malvern, UK) or a BI-200SM Research Goniometer Light Scattering system (Brookhaven Instruments, Holtsville, NY, USA). Mean vesicle diameters were typically ∼120 nm (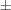
5 nm).

Donor multilamellar vesicles (20 mg ml^−1^ total lipid concentration) composed of the outer leaflet lipids were prepared by hydrating the dry lipid films in water containing 20%(*w*/*w*) sucrose using vortex mixing in combination with three freeze/thaw cycles. Donor MLVs were then diluted 20-fold with water and centrifuged for 30 min at 20 000 *g* in order to remove extravesicular sucrose. The resulting pellet was re-suspended in a 35 m*M* mβCD solution at a lipid:mβCD ratio of 1:8 and incubated for 2 h at room temperature, while being gently stirred.

Lipid exchange was initiated by mixing acceptor and donor vesicle suspensions (donor/acceptor ratio 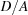 = 2 for POPC aLUVs and 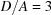 for DPPC/POPC aLUVs) and allowed to proceed for 1 h at room temperature. The resultant aLUVs were diluted eightfold with water and then separated from the donor MLVs *via* centrifugation at 20 000 *g* for 30 min. The supernatant containing the aLUVs (as well as residual CD and sucrose) was then concentrated to <0.5 ml with a centrifugal ultrafiltration device (100 kDa molecular weight cutoff, 11 ml volume, 5000 *g*). The initial concentration step was followed by three cycles of successive dilution to 11 ml and concentration to <0.5 ml, effectively removing residual CD and sucrose, and allowing for the exchange of H
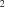
O with D
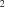
O for SANS and 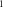
H NMR experiments. The mean diameter of the aLUVs was ∼120 nm (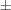
5 nm), a diameter (within measurement uncertainty) identical to that of the acceptor LUVs prior to exchange. Lipid exchange efficiency and inner/outer leaflet distribution were determined by gas chromatography and mass spectrometry (GC–MS), or ultra performance liquid chromatography and mass spectrometry (UPLC–MS), combined with 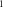
H NMR measurements, as detailed by Heberle *et al.* (2016 ▸) and in the supporting information. We demonstrated previously that membrane structural parameters are not altered by this preparation (Heberle *et al.*, 2016 ▸).

In some cases, symmetric LUVs were prepared from aLUVs by chemical scrambling as follows. aLUVs were dried to a film under reduced atmospheric pressure using a rotary evaporator with the water bath set to 303–323 K. The dried film was then redissolved in chloroform. From that point on, the sample preparation was identical to that of the acceptor LUVs, as described above. We refer to these LUVs as ‘scrambled’ vesicles throughout the text.

### 3.2. Small-angle neutron scattering   

Neutron scattering experiments were performed at the BL-6 extended-*Q*-range small-angle neutron scattering (EQ-SANS) instrument of the Spallation Neutron Source, located at Oak Ridge National Laboratory (ORNL), and KWS-1 at the FRM II reactor (Munich–Garching, Germany) (Frieling­haus *et al.*, 2015 ▸; Feoktystov *et al.*, 2015 ▸). Samples were loaded into 1 or 2 mm path length quartz banjo cells or 1 mm path length 404 000-QX quartz cuvettes (Hellma, Jena, Germany), and mounted in a temperature-controlled cell holder with ∼1 K accuracy. Typical measurement times were 30 min. EQ-SANS data were taken at two sample-to-detector distances (SDDs), 1.3 and 4.0 m, using wavelength bands of 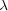 = 4.0–7.5 Å and 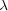 = 10.0–13.5 Å, respectively, corresponding to scattering vector magnitudes of *q* = 0.005–0.5 Å^−1^. Data were collected with a two-dimensional 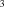
He position-sensitive detector and reduced to one-dimensional *I*(*q*) scattering curves using *Mantid* (Arnold *et al.*, 2014 ▸). KWS-1 data were obtained with a two-dimensional scintillation detector using neutrons of 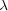 = 5 Å (wavelength spread FWHM: 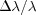 = 0.1) and two SDDs, 1.21 and 7.71 m, yielding a *q* range of 0.005–0.42 Å^−1^. Data were corrected for detector pixel sensitivity, dark current, sample transmission and background scattering from D
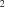
O using the *QTIKWS* software from JCNS (Garching, Germany).

### 3.3. Small-angle X-ray scattering   

SAXS data for POPC aLUVs were collected at the P12 BioSAXS beamline, located at the storage ring PETRA III (EMBL/DESY) in Hamburg, Germany (Blanchet *et al.*, 2015 ▸). This beamline delivers a total photon flux of 5 × 10^12^ s^−1^ focused to a spot of 120 × 200 µm (full width at half-maximum). The combination of 20 keV (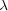 = 0.6 Å) photons and SDD = 3.1 m yielded an accessible *q* range of 0.04–0.92 Å^−1^. A Pilatus 2M detector (Dectris, Switzerland) was used for data collection. SAXS data from DPPC/POPC aLUVS were obtained at the ESRF BM29 BioSAXS beamline (Pernot *et al.*, 2013 ▸) (Grenoble, France) using a Pilatus 1M detector (Dectris, Switzerland), with 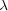 = 0.99 Å and SDD = 2.869 m, yielding an accessible *q* range of 0.003–0.5 Å^−1^. At both beamlines, samples were transferred prior to measurement into multi-well plates and equilibrated for 10 min in a temperature-controlled block. An automated system delivered 20–35 µl of the lipid sample into a preheated glass capillary. For each sample, 20 (P12) or ten (BM29) frames were recorded with an exposure time of 0.095 s (P12) or 0.5 s (BM29). The water background was measured before and after each sample. To determine the occurrence of possible radiation damage, data collected in subsequent frames were compared by a standard F-test (Petoukhov *et al.*, 2007 ▸). Data treatment was performed using the *ATSAS* suite (Petoukhov *et al.*, 2012 ▸).

## 4. Models   

It was shown previously (Kiselev *et al.*, 2002 ▸; Pencer *et al.*, 2006 ▸) that coherent scattering from symmetric LUVs can be approximated under certain conditions by 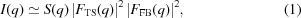
where 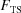 is the form factor of a thin spherical shell (containing information about vesicle size and polydispersity), 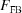 is the form factor of a flat bilayer sheet (containing information about the distribution of matter across the bilayer) and *S* is the interparticle structure factor (accounting for interactions between the particles, and equal to unity for a sufficiently diluted system). Equation (1) is often referred to as the separated form factor (SFF) approximation and is valid when the length scales of vesicle radius and bilayer thickness are well separated, such that 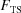 and 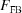 can be treated independently (Pencer *et al.*, 2006 ▸). As shown by the same authors, ∼5 nm thick bilayers and vesicles larger than 50 nm have negligible contributions to 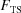 for 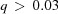
 Å^−1^, and the scattered intensity can be approximated by 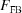 only.

Brzustowicz & Brunger (2005 ▸) demonstrated that, for freely floating vesicles with transmembrane asymmetry, a flat bilayer model provides a good description of the scattered intensity. This enables us to apply the SFF method to aLUV data, with the caveat that inner and outer membrane leaflets cannot be unambiguously assigned without *a priori* knowledge of the membrane’s composition. In our case, leaflet compositions were obtained independently using solution NMR in combination with GC–MS or UPLC–MS [see Heberle *et al.* (2016 ▸) and supporting information §1].

The flat bilayer form factor can be expressed as 
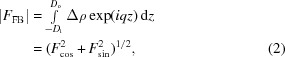
where 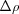 is the difference between the SLDs of the membrane and the solvent, and 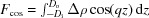 and 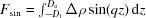 are the real and imaginary parts of 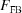. The integral extends over the full bilayer thickness, that is from its innermost distance 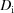 to its outermost distance 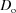.

### 4.1. Asymmetric slab models   

#### 4.1.1. Four-slab model   

**Figure 1:**
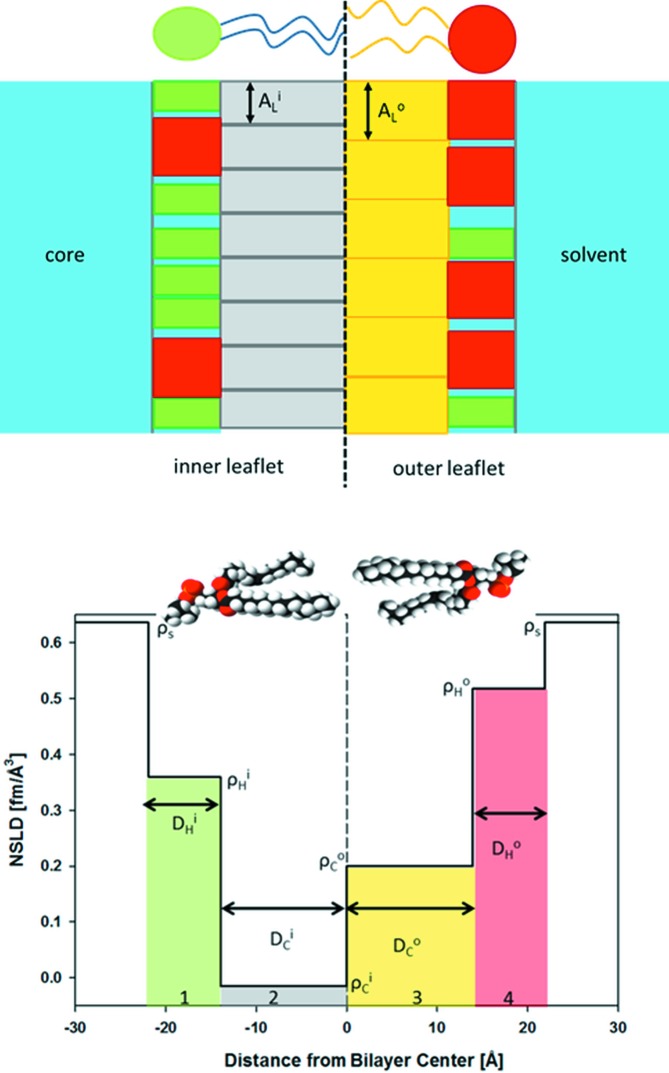
Schematic illustration of the four-slab model. Upper, space-filling representation of an asymmetric bilayer. Lower, the neutron scattering length density (NSLD) profile across the bilayer is obtained by averaging the composition of each slab.

The four-slab model has been used previously for analyzing aLUV SANS data (Heberle *et al.*, 2016 ▸). For completeness, we summarize its main features below. The bilayer’s SLD profile is given by 
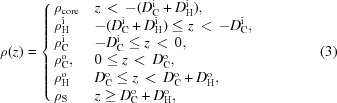
where ρ are SLDs and *D* are the thicknesses of the individual slabs. The superscripts o and i denote outer and inner leaflets, respectively (see also Fig. 1 ▸). If the SLD of the membrane core matches that of the solvent (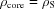), the limits of the integral in equation (2) are well defined, yielding 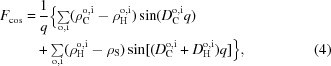
where 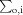 denotes the sum over either all outer or all inner leaflet parameters (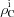, 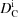, 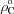, 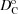, 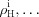), respectively, and 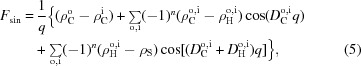
where 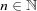 and
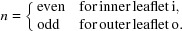



Following the approach used by Kučerka and co-workers (Kučerka *et al.*, 2004 ▸, 2008 ▸; Kučerka, Pencer, Nieh & Katsaras, 2007 ▸), it is possible to reduce the number of adjustable parameters by enforcing matter conservation (*i.e.* by assuming volume incompressibility and space filling), which essentially couples the thicknesses of the individual layers to the projected area per lipid 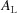 and lipid molecular volume 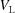 (Fig. 1 ▸ upper panel). However, we must first consider that aLUVs will differ in the type and number of lipids in the outer and inner leaflets. This is accounted for by introducing different leaflet molar ratios 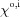 for the inner and outer leaflet lipids. Small differences between the surface areas of the inner and outer leaflets lead to an additional scaling of 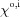, as detailed in the supporting information (§2). The number of headgroup-bound water molecules, 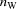, will also in general be different for each leaflet. The average molecular volumes of the lipid headgroup and hydrocarbon layers are then calculated as mole-fraction-weighted sums: 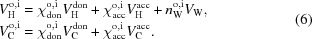
Similarly, the corresponding average coherent neutron scattering lengths are given by 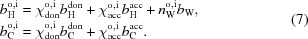
Super/subscripts ‘don’ and ‘acc’ differentiate, respectively, between donor and acceptor lipids, 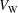 is the molecular volume of water, 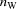 is the number of bound water molecules, and 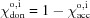. Lipid volumes can be determined by either independent experiments (Greenwood *et al.*, 2006 ▸; Hodzic *et al.*, 2008 ▸; Murugova & Balgavý, 2014 ▸) or atomistic simulation (Petrache *et al.*, 1997 ▸). For the present work we used volumes determined experimentally by densitometry and reported by Kučerka *et al.* (2011 ▸). Hence, the ρs and *D*s in equation (4) can be replaced by 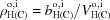 and 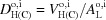, reducing the number of adjustable parameters to four (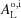, 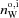). In this work, 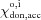 was independently determined by GC–MS and NMR analysis of the aLUV composition for each sample (supporting information). Alternatively, 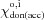 can be a free parameter if it is unknown, or constrained in order to account for any uncertainty in its determination by analytical methods.

A complication we encountered was that the different contrast aLUV preparations showed a small but non-negligible variation of outer leaflet exchange (see *e.g*. Table S6). In order to account for this, we approximated the 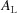 in each leaflet by a composition-weighted sum of the areas of its constituent lipids: 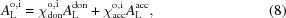
where the lipid areas of donor and acceptor lipids 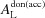 are now adjustable parameters. Finally, we defined the hydrocarbon chain length of each leaflet as 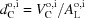 and the total bilayer (Luzzati) thickness as 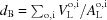 (Nagle & Tristram-Nagle, 2000 ▸).

#### 4.1.2. Six-slab model   

**Figure 2:**
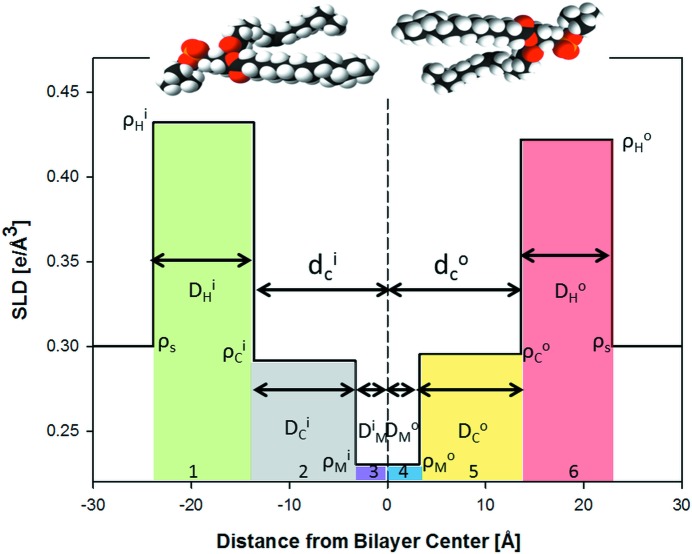
Schematic illustration of the six-slab electron density profile model.

For SAXS analysis, an additional slab for the terminal methyl group of each leaflet must be added owing to the significant differences in electron densities between CH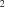 and CH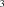 groups, resulting in a six-slab model for the electron density profile: 
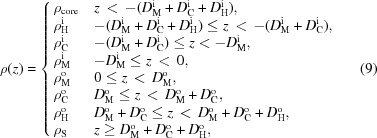
where subscripts ‘M’ denote the central methyl slabs (Fig. 2 ▸).

From equation (9) we calculate the real and imaginary parts of the form factor: 
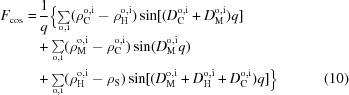
and 
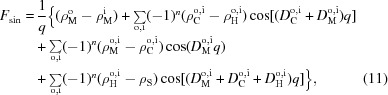
where 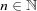, 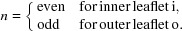



Using similar arguments as in §4.1.1, the electron densities and slab thicknesses can be replaced by 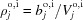 and 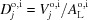, where 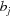 now refers to the number of electrons for each slab 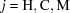. We further parsed the headgroup and methyl slabs to account for their contributions to the neighboring hydrocarbon methylene region: 
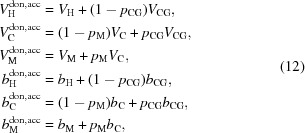
where 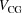 and 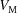 are the respective volumes of the carbonyl-glycerol (CG) and methyl groups, and 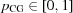 distributes the lipid’s CG contributions between the headgroup and hydrocarbon regions (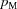 does the same for methyl slabs). Volumes and electron densities are then calculated according to equations (6) and (7), where 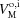 and 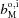 are determined analogously to 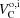 and 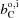.

Volumetric data for the individual slabs were taken from the literature (Kučerka *et al.*, 2011 ▸). Temperature-dependent values of 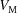 can be found, for example, in the work of Small (1986 ▸), Koenig & Gawrisch (2005 ▸) and Kučerka *et al.* (2011 ▸). Depending on whether 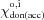 is known or not, the six-slab model has either six (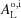, 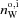, 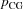, 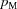) or eight adjustable parameters. Finally, we note that the electron density contrast between acyl chains of the inner and outer leaflets is generally weak. However, this contrast can in principle be enhanced by introducing brominated or fluorinated lipids into one of the leaflets (McIntosh *et al.*, 1996 ▸; Hristova & White, 1998 ▸).

### 4.2. SDP model for asymmetric membranes   

**Figure 3:**
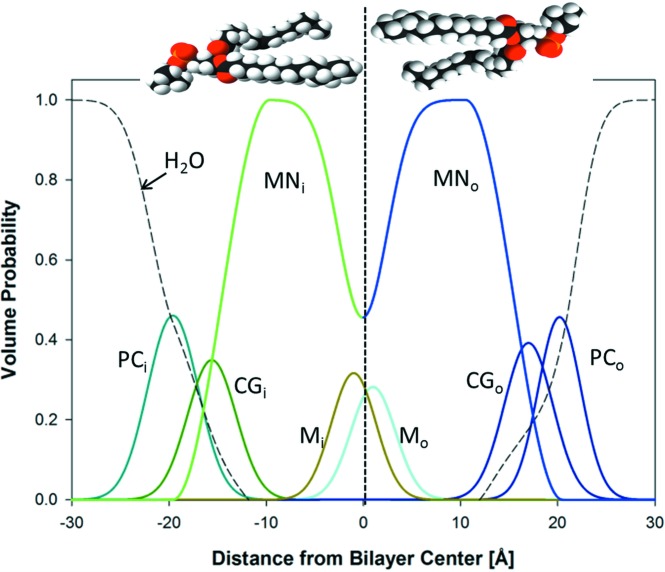
Schematic illustration of the volume probability distribution for an asymmetric bilayer.

The SDP model describes the bilayer structure in terms of one-dimensional volume probability profiles (VPPs) of quasi-molecular lipid fragments. The VPPs are scaled by either the fragment’s total coherent neutron scattering length (in the case of SANS) or the number of electrons (in the case of SAXS) to obtain the SLD profile (Pabst *et al.*, 2010 ▸; Marquardt, Heberle *et al.*, 2015 ▸). Inspired by Kučerka, Pencer, Sachs *et al.* (2007 ▸), we parse each leaflet of the asymmetric bilayer as follows: choline methyl + phosphate + CH
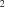
CH
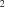
N (PC); carbonyl + glycerol (CG); hydrocarbon (HC); and methyl (M) groups. The volume probabilities for the PC, CG and M groups are modeled as Gaussians:
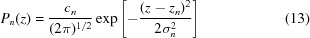
for *n* = PC, CG, M, where 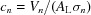 and 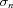 and 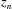 are the width and position of the distribution, respectively (Fig. 3 ▸). The HC groups are described by smooth plateau-like functions using error functions (Pabst *et al.*, 2010 ▸). However, our modeling must also account for the different contrasts in the inner and outer HC layers, which require two separate smooth bridging functions, leading to a significant increase in computational resources. We therefore applied [following Wiener *et al.* (1989 ▸)] a half-period squared sine/cosine function: 
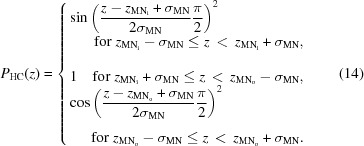
Here, 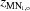 is the 0.5-probability value for the hydrocarbon region (and thus also defines the thickness of the inner and outer leaflet hydrocarbon regions 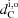, in accordance with its definition as a Gibbs dividing surface) and 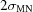 is the width of the squared sine/cosine functions. The probability function of the methylene regime is 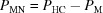.

We note two additional modifications to the SDP description for symmetric bilayers. Firstly, the choline and phosphate groups are combined into a single Gaussian in order to decrease the number of fitting parameters. The ensuing decrease in structural resolution is, however, within experimental error, as determined from fits of previously reported POPC form factors (Kučerka *et al.*, 2011 ▸) using either combined or separate headgroup Gaussians. Secondly, in some cases, two distinct methyl groups must be modeled, for example when the outer and inner bilayer leaflets contain contrasting hydrocarbon chains. This is achieved by displacing each leaflet’s methyl group slightly from the bilayer midplane, ensuring, however, that their combined envelope function is a single Gaussian, as observed in symmetric bilayers when both amplitudes are equal (Fig. 3 ▸).

The water (solvent) probability function 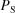 is defined as 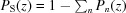, with 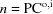, 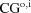, 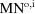, 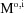 (Klauda *et al.*, 2006 ▸; Kučerka, Pencer, Sachs *et al.*, 2007 ▸).

For the real part of 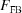 we then calculate 
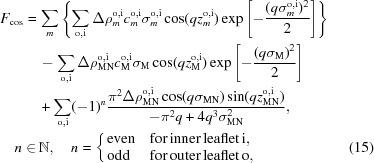
where 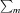 denotes the sum over the PC, CG and M groups. For the imaginary part of F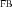 we calculate 
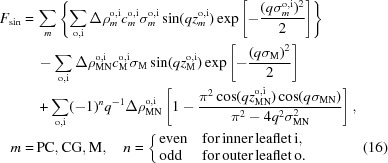



Several additional constraints were imposed during the fitting procedure. On the basis of previous results showing that the distance 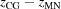 between the carbonyl-glycerol group and the hydrocarbon/headgroup interface lies between 0.45 and 1.1 Å for different PC lipids (Kučerka *et al.*, 2011 ▸), we fixed this value to 1 Å. Additionally, the locations of the methyl groups were fixed at a distance of 1 Å from the bilayer center, *i.e.* 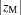 = 1 Å, and 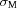 was set to the value obtained by fitting the sum of inner and outer leaflets 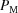 to the envelope function given for the corresponding symmetric bilayers (Kučerka *et al.*, 2011 ▸). This yielded 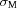 = 2.38 Å for DPPC and 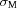 = 2.02 Å for POPC bilayers. Finally, as mentioned, our aSDP model combines the choline and phosphate groups into a single Gaussian probability function. However, in order to obtain reasonable 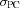 values, the form factors reported for POPC and DPPC by Kučerka *et al.* (2011 ▸) were refitted with our aSDP model.

After these constraints were applied, six adjustable parameters remained for the aSDP model: 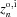 and 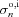, where *n* = PC, CG, MN (if needed 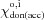 can also be varied). In order to account for variations in outer lipid exchange efficiency, when jointly analyzing different contrast data sets obtained from different physical samples, these parameters can be rewritten as a weighted sum of values for the individual donor and acceptor lipid species: 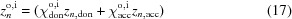
and 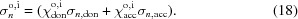



To increase the stability of the fits, we derived the individual 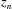 and 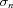 values of the acceptor and donor lipids by scaling their reported values in single-component bilayers (Kučerka *et al.*, 2011 ▸). For example, 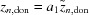 and 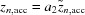, 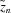 being the reported literature value, and 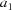 and 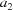 being the fitted scaling parameters. The observed variations in 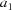 and 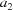 were between 0.96 and 1.04. Structural parameters were calculated analogously to the slab model using 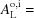 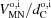, where 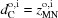, and 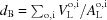.

### 4.3. Joint analysis of SANS and SAXS data   

In order to fully exploit the benefits of contrast variation, all SANS and SAXS data were fitted simultaneously in a joint analysis taking into account the appropriate experimental resolution (see *e.g.* Feigin & Svergun, 1987 ▸). In the case of the asymmetric slab models, this was achieved by requiring common values for 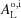 and 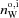 for all data sets. For the aSDP model, the volume probability distributions of quasi-mol­ecular fragments serve as a common backbone for a joint SANS/SAXS data analysis. The applied optimization function 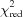 included all SANS and SAXS data sets for a given aLUV system. Specific weighting schemes took into account the importance of matching the first minimum displayed in the SANS data, as well as the SAXS intensity modulations at high *q* vector magnitudes. This was achieved by decreasing the experimentally determined uncertainties in these regions by a factor of 0.1–0.5. Further, the SAXS data were weighted by a factor of 0.5 with respect to the SANS data. The reported 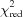 values were recalculated after releasing all constraints and weights to avoid any influence from the specific weighting.

Different optimization routines were also applied. In the case of the asymmetric slab model the small number of free parameters allowed us to apply the trust region reflective algorithm, which is similar to the Levenberg–Marquardt algorithm, but with a restricted step size, thereby preventing it from overstepping (Yuan, 2000 ▸). Because of the large number of adjustable parameters in the aSDP model, there is an inherent danger that a deterministic search algorithm (such as the one used for the asymmetric slab model) will become trapped in a local minimum. In this case, random search or stochastic algorithms, such as the differential evolution (DE) algorithm (Price *et al.*, 2006 ▸; Storn & Price, 1997 ▸; Price & Storn, 1997 ▸), offer a different strategy. For example, the DE algorithm performs a global search for the best solution starting from an initial population of solutions; these are subsequently combined and/or ‘mutated’ to form new solutions that are accepted or rejected on the basis of their agreement with experimental data.

The uncertainties of the joint SAXS/SANS analysis were determined to be <2% for symmetric systems and <3% for asymmetric systems. These values were estimated by a variation of initial (starting) parameters, number of iterations and termination tolerances.

## 5. Results and discussion   

### 5.1. Testing models on symmetric LUVs   

**Figure 4:**
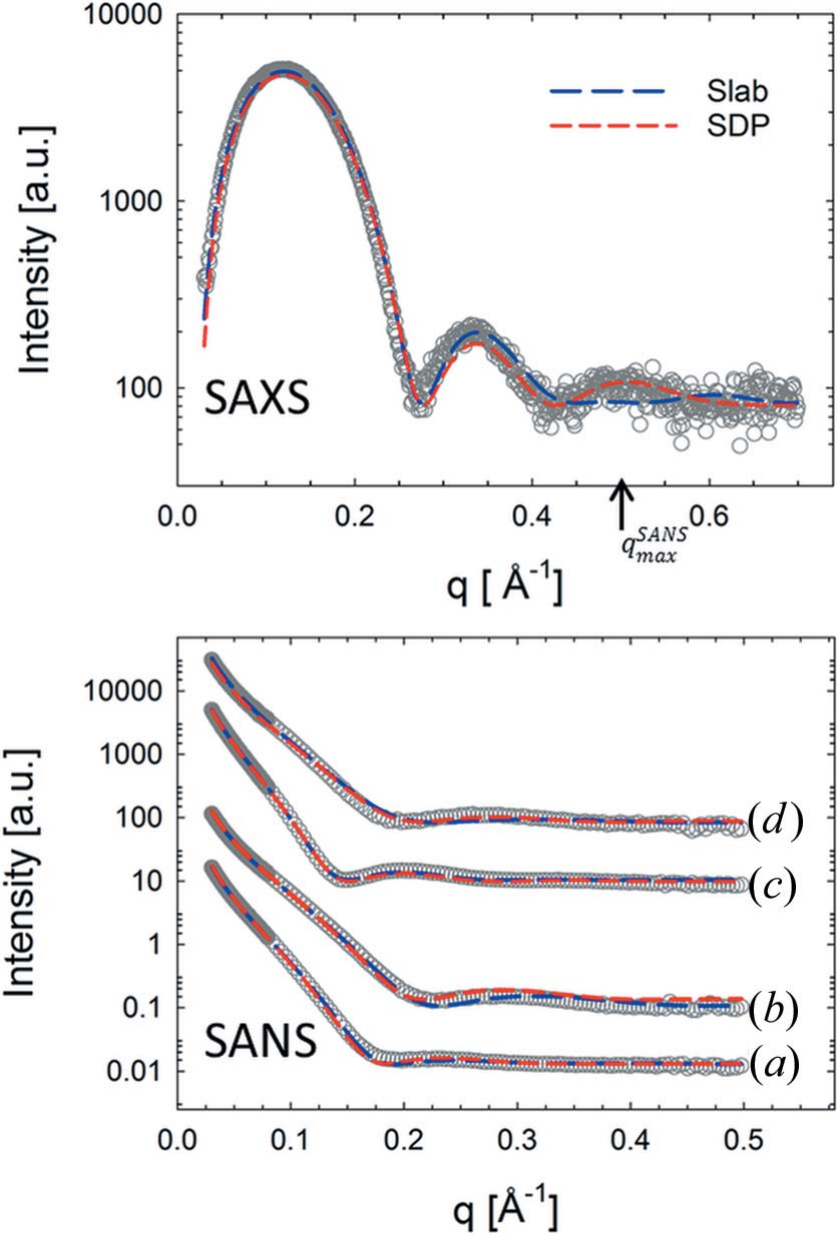
Joint analysis of SAXS (top panel) and SANS (bottom panel) data from symmetric POPC LUVs with different internal contrasts, namely (*a*) POPC, (*b*) POPC-d13, (*c*) POPC-31 and (*d*) POPC-d44 (*T* = 293 K). The maximum SANS resolution in reciprocal space is indicated by the 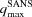 arrow in the SAXS panel. Dashed lines are best fits using the asymmetric slab (blue long-dashed line) and SDP models (red short-dashed line). Data are offset vertically for clarity. The SANS data in the lower panel were previously published by Heberle *et al.* (2016 ▸) and are reanalyzed here.

All models were assessed for their ability to reproduce previously reported structural data for symmetric bilayers. To this end, we prepared four symmetric LUV samples with different internal contrasts (*i.e.* POPC, POPC-d13, POPC-d31 and POPC-d44). SANS data were taken from the work of Heberle *et al.* (2016 ▸) and reanalyzed jointly with new data from SAXS experiments. In the analyis of these datasets, we constrained 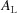 and 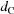 from the inner and outer leaflets to be identical. The corresponding SANS and SAXS data and their fits are shown in Fig. 4 ▸. Structural parameters determined from the joint analysis (Table 1 ▸) were also compared with results obtained by analyzing each dataset individually. The latter comparison shows that 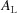 obtained from standalone SAXS data was smaller than that obtained from standalone SANS data, while 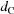 and 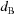 were larger for the SAXS analysis than for the SANS analysis owing to the inverse relationship between lipid area and bilayer thickness. The jointly analyzed values are, however, closer to those obtained by SANS, which can be understood by the fact that the applied definitions for 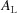, 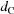 and 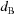 depend on the position of the glycerol backbone, to which neutrons are most sensitive. Furthermore, the slab and SDP models yielded practically identical values for 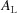 and 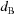 when all data sets were included. In terms of fit quality, 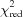 were generally smaller for the SDP model, which we attribute to the model’s higher intrinsic resolution. Note that the high 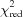 values of SANS are due to the four different contrasts that were fitted simultaneously.

It is instructive to compare our results with the literature values listed in Table 1 ▸. Within experimental uncertainty, we find good agreement with the results of Kučerka *et al.* (2011 ▸), who also applied an SDP-based analysis similar to ours but who did not use lipid isotopes, and a re-evaluation of these data in terms of an atomistic model (Fogarty *et al.*, 2015 ▸).

### 5.2. Testing the models using isotopic aLUVs   

We next analyzed aLUVs composed of different POPC isotopes in the inner and outer leaflets, *i.e.* POPC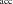/POPC-d44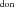 and POPC-d44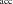/POPC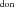 for SANS and POPC-d13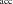/POPC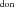 for SAXS. This labeling scheme allowed us to unambiguously resolve the structure of the inner and outer bilayer leaflets. For both samples, we achieved approximately 60% exchange of the outer leaflet lipids (see Table S3 for details). SANS data previously reported by Heberle *et al.* (2016 ▸) were reanalyzed together with new SAXS data using both models.

**Figure 5:**
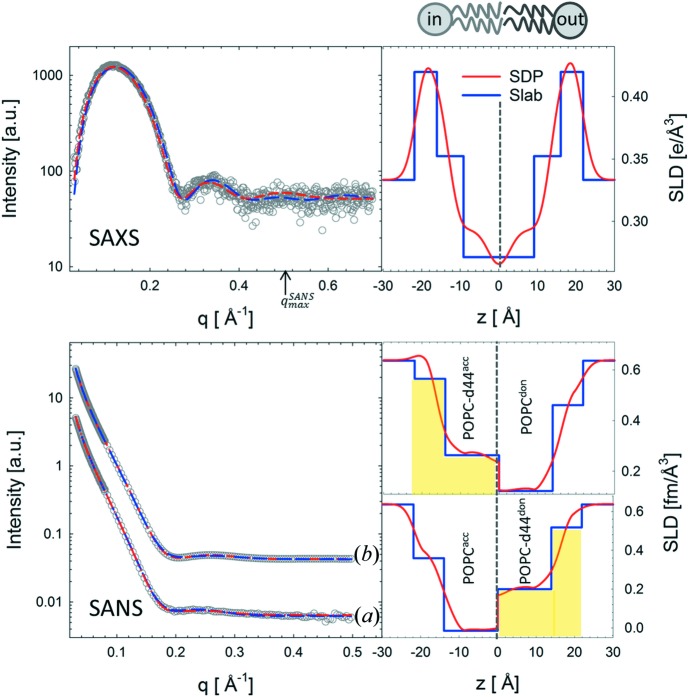
Structural parameters for POPC aLUVs measured at 293 K. Top panel, SAXS data (open circles) from POPC-d13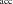/POPC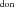 aLUVs, and corresponding fits (dashed lines) and electron density profiles (right). The maximum SANS resolution in reciprocal space is indicated by the 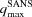 arrow. Bottom panel, SANS data (open circles) from (*a*) POPC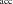/POPC-d44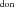 and (*b*) POPC-d44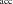/POPC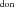 aLUVs and corresponding fits (dashed lines), and neutron scattering length density profiles (right). Blue and red colors denote analysis using the slab and SDP models, respectively. Structural parameters are listed in Table 2 ▸. The SANS data in the lower panel were previously published by Heberle *et al.* (2016 ▸) and are reanalyzed here.

Fig. 5 ▸ shows the corresponding SAXS and SANS data, and their fits obtained from joint analysis. Results of the structural parameters are presented in Table 2 ▸. On average, the structural parameters are, within experimental uncertainty, equal to those obtained for symmetric LUVs. This is consistent with our previous finding that the aLUV preparation does not alter the membrane structure (Heberle *et al.*, 2016 ▸). Analysis using the slab model yielded larger values for 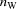 and 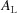 for the inner leaflet compared to the outer leaflet. This result appears to be physically unrealistic, considering that previous studies found that membrane curvature induces a greater packing density (smaller 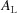) for inner leaflet lipids (Huang & Mason, 1978 ▸; Smolentsev *et al.*, 2016 ▸). We therefore constrained the inner leaflet 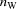 and 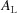 values not to exceed the outer leaflet values, which resulted in both leaflets having identical values for these parameters. An alternative interpretation is that inner leaflet lipids may protrude out of the membrane to avoid lateral compression, leading to a rougher inner surface (Brzustowicz & Brunger, 2005 ▸) and to 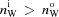, which can then result in 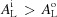. Comparing the 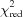 values of the constrained and unconstrained fits (Table 2 ▸), as well as fit residuals (Fig. S1), we conclude that these scenarios cannot be distinguished owing to the inherent resolution limitations of the slab model.

In the case of the aSDP model, unconstrained fits led to 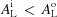, and consequently to 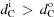. To further understand the coupling of vesicle size/curvature to lipid packing differences of the inner and outer leaflets, additional studies would be needed that are beyond the scope of the current work. We note that 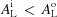 is consistent with an earlier analysis of 20 nm diameter vesicles (Huang & Mason, 1978 ▸), which because of their smaller radius resulted in significant differences between 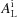 and 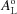. However, 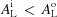 was also suggested for 100 nm vesicles in a recent spectroscopic study (Smolentsev *et al.*, 2016 ▸).

### 5.3. DPPC/POPC asymmetric membranes   

**Figure 6:**
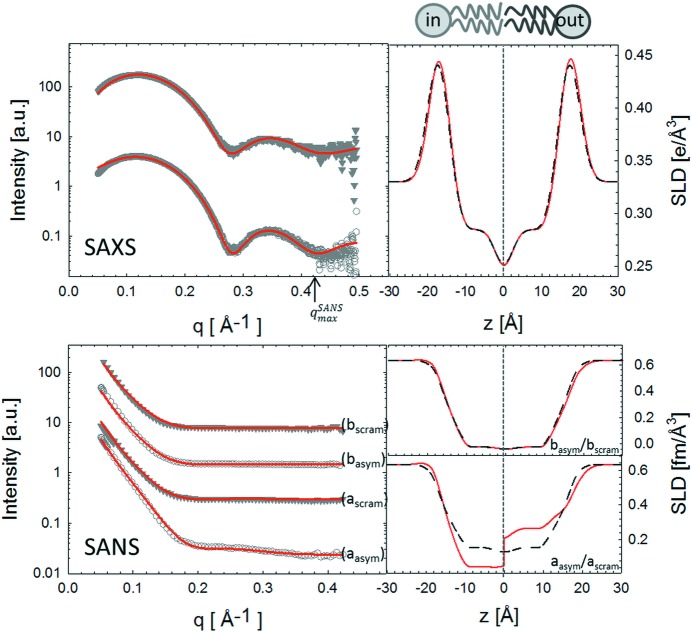
SDP analysis (red lines) of DPPC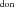/POPC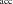 aLUVs (open circles) and scrambled LUVs (filled triangles). The panels on the right show the corresponding SLDs (red: aLUVs; dashed black: scrambled LUVs). The different contrast samples for SANS experiments were DPPC-d62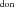/POPC-d13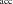 (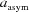/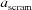) and DPPC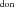/POPC-d13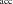 (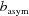/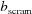). The maximum SANS resolution in reciprocal space is indicated by the 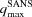 arrow in the SAXS panel. Data are offset vertically for clarity.

Fig. 6 ▸ shows the aSDP analysis of DPPC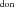/POPC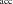 aLUVs using two different contrasts (*i.e.* DPPC-d62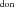/POPC-d13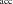 and DPPC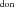/POPC-d13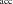). The analysis also included scrambled DPPC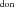/POPC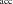 vesicles (see §3) in order to examine the impact of transbilayer lipid asymmetry on structure. Data analyzed in terms of the slab model are presented in Fig. S2. A previous analysis of outside gel/inside fluid DPPC/POPC aLUVs at room temperature showed a partial fluidization of the outer leaflet, observed as a significantly larger 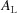 as compared to typical gel-phase values (Heberle *et al.*, 2016 ▸). In order to determine whether such a transbilayer coupling persists in fluid aLUVs, we carried out experiments at 323 K, *i.e.* above the melting temperature of both lipids.

Analysis of the aLUV composition yielded significant differences in samples with different contrasts (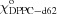 = 0.35–0.45 and 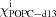 = 0.85–0.92, see Table S6). Data were therefore analyzed according to equations (8), (17) and (18), *i.e.* in terms of the individual structures of DPPC and POPC lipids.

The results from this analysis (Table 3 ▸) show good agreement for the different structural parameters of asymmetric and scrambled samples. The areas for DPPC (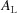 = 63.1 Å^2^) and POPC (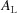 = 67.3 Å^2^) were also consistent with those from a previous analysis of single-component bilayers at 323 K (Kučerka *et al.*, 2011 ▸). Because the differences between structural parameters obtained in aLUVs and those reported for corresponding single-component bilayers are within experimental uncertainties, we conclude that there is no transbilayer structural coupling in DPPC/POPC aLUVs when both leaflets are in the fluid phase. However, we cannot exclude the possibility of transbilayer coupling in fluid membranes with a higher outer leaflet exchange and/or for different lipid systems. We further note that there is an overall good agreement between the results obtained through the slab and aSDP models.

In order to examine leaflet-specific structural features of DPPC/POPC aLUVs, we calculated the molecular averages of 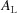, 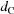 and 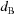 according to the aLUV compositions (Table 4 ▸). Compared to scrambled LUVs, 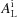 is slightly larger than 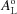, leading to 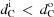. This is in contrast to our findings for POPC aLUVs (Table 2 ▸), where the inner leaflet was somewhat more densely packed than the outer leaflet. This result was not unexpected, however, since POPC has a larger area per lipid than DPPC at 323 K (Kučerka *et al.*, 2011 ▸; Fogarty *et al.*, 2015 ▸) (see also Table 3 ▸). The inner leaflet – which is essentially pure POPC – thus has a larger average 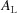 than the outer leaflet, which contains a substantial amount of DPPC in addition to POPC.

## 6. Conclusion   

We have adapted a low-resolution slab model and a high-resolution SDP model for the joint SANS/SAXS analysis of asymmetric lipid vesicles. These new models provide analytical tools to study putative interleaflet coupling mechanisms in asymmetric bilayers, which better represent the asymmetry found in most biological membranes.

Application of the aSDP model requires a large number of adjustable parameters, some of which were constrained in order to avoid nonphysical results (see also *e.g.* Table S5). Additionally, we applied a DE algorithm to prevent the optimization routine from becoming trapped in local minima. In the case of the slab model, which has fewer free parameters, the trust region reflective algorithm was sufficient. On the basis of the fit quality, as judged by 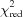, the extra computational effort required by the aSDP model is justified. However, in cases where only SANS data are available, the slab model may provide a more reliable analysis. In order to apply this model to SAXS data, an additional slab for the methyl groups is needed.

Both models were tested on isotopically labeled variants of POPC and DPPC/POPC aLUVs. Interestingly, our analysis of POPC aLUVs suggested 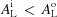, consistent with the presence of residual curvature strain in our 120 nm diameter aLUVs. Furthermore, in the case of fluid DPPC/POPC aLUVs at 323 K, we did not find transbilayer coupling of the individual leaflet structures. We believe that, together with the recently reported protocol for preparing aLUVs (Heberle *et al.*, 2016 ▸), this work sets the stage for future studies of multicomponent aLUVs (*e.g.* including cholesterol) that are needed to understand the complex structure of asymmetric membranes on the sub-nanometre level.

## 7. Related literature   

For further literature related to the supporting information, see Hartler *et al.* (2011 ▸), Knittelfelder *et al.* (2014 ▸) and Perly *et al.* (1985 ▸).

## Supplementary Material

Supporting information file. DOI: 10.1107/S1600576717000656/vg5060sup1.pdf

## Figures and Tables

**Table 1:** Structural parameters of DPPC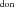/POPC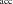 aLUVs and LUVs obtained after scrambling Parameter uncertainties are estimated to be <2%.

Model	*A*_L_ (Å^2^)	*d*_C_ (Å)	*d*_B_ (Å)	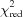
Slab 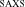 †	65.3	14.0	38.2	1.1
Slab 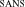 ‡	67.5	13.6	36.9	94.5
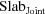 §	67.5	13.6	36.9	54.2
SDP 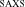 †	63.7	14.4	39.2	0.6
SDP 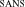 ‡	66.8	13.7	37.3	142.2
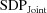 §	66.3	13.8	37.6	34.6
SDP 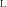 ¶	62.7	14.6	39.8	–
ADP 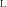 ††	67.0	13.7	37.2	–

† Analysis of SAXS data only.

‡ Joint analysis of different contrast SANS data only. SANS data were previously published by Heberle *et al.* (2016 ▸) and are reanalyzed here.

§ Joint analysis of SANS and SAXS data.

¶ From Kučerka *et al.* (2011 ▸).

†† From Fogarty *et al.* (2015 ▸).

**Table 2:** Structural parameters of POPC aLUVs Values in parentheses for the slab model correspond to results obtained upon removing area constraints. Parameter uncertainties are estimated to be <3%.

	*A*_L_ (Å^2^)		*d*_C_ (Å)			
Model	Outer	Inner	Outer	Inner	*d*_B_ (Å)	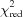
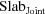 †	65.5 (63.8)	65.5 (67.8)	14.0 (14.4)	14.0 (13.5)	38.0 (37.9)	7.0 (6.3)
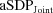 †	65.7	63.4	14.0	14.4	38.6	6.6

† Joint analysis of SANS and SAXS data. The SANS data were previously published by Heberle *et al.* (2016 ▸) and are reanalyzed here.

**Table 3:** Structural parameters of DPPC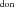/POPC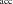 aLUVs and LUVs obtained after scrambling Parameter uncertainties are estimated to be <3%.

	*A*_L_ (Å^2^)		*d*_C_ (Å)	
Model (sample)	DPPC	POPC	DPPC	POPC
Slab (aLUV)	61.7	68.7	14.5	13.7
aSDP (aLUV)	62.6	67.9	14.3	13.9
Slab (LUV†)	64.9	67.8	13.8	13.8
SDP (LUV†)	64.9	69.1	13.8	13.7

† Scrambled.

**Table 4:** Structural parameters averaged over different contrasts obtained from SDP analysis of DPPC/POPC aLUVs Parameter uncertainties are estimated to be <3%.

	*A*_L_ (Å^2^)		*d*_C_ (Å)		
System	Outer	Inner	Outer	Inner	*d*_B_ (Å)
aLUVs	65.7	67.2	14.1	14.0	38.0
Scrambled	67.8	67.8	13.9	13.9	37.2

## References

[bb1] Arnold, O. *et al.* (2014). *Nucl. Instrum. Methods Phys. Res. Sect. A*, **764**, 156–166.

[bb2] Blanchet, C. E., Spilotros, A., Schwemmer, F., Graewert, M. A., Kikhney, A., Jeffries, C. M., Franke, D., Mark, D., Zengerle, R., Cipriani, F., Fiedler, S., Roessle, M. & Svergun, D. I. (2015). *J. Appl. Cryst.* **48**, 431–443. 10.1107/S160057671500254X PMC4379436 25844078

[bb3] Brzustowicz, M. R. & Brunger, A. T. (2005). *J. Appl. Cryst.* **38**, 126–131.

[bb4] Cheng, H.-T., Megha & London, E. (2009). *J. Biol. Chem.* **284**, 6079–6092. 10.1074/jbc.M806077200 PMC2649079 19129198

[bb5] Chiantia, S. & London, E. (2013). *Encyclopedia of Biophysics*, pp. 1250–1253. London: Springer.

[bb6] Devaux, P. F. (1991). *Biochemistry*, **30**, 1163–1173. 10.1021/bi00219a001 1991095

[bb7] Feigin, L. A. & Svergun, D. I. (1987). *Structure Analysis by Small-Angle X-ray and Neutron Scattering.* New York: Plenum Press.

[bb8] Feoktystov, A. V., Frielinghaus, H., Di, Z., Jaksch, S., Pipich, V., Appavou, M.-S., Babcock, E., Hanslik, R., Engels, R., Kemmerling, G., Kleines, H., Ioffe, A., Richter, D. & Brückel, T. (2015). *J. Appl. Cryst.* **48**, 61–70.

[bb9] Fogarty, J. C., Arjunwadkar, M., Pandit, S. A. & Pan, J. (2015). *Biochim. Biophys. Acta*, **1848**, 662–672. 10.1016/j.bbamem.2014.10.041 25448879

[bb10] Frielinghaus, H., Feoktystov, A., Berts, I. & Mangiapia, G. (2015). *J. Large-Scale Res. Facilities*, **1**, 28.

[bb11] Greenwood, A. I., Tristram-Nagle, S. & Nagle, J. F. (2006). *Chem. Phys. Lipids*, **143**, 1–10. 10.1016/j.chemphyslip.2006.04.002 PMC2695672 16737691

[bb50] Hartler, J., Trotzmuller, M., Chitraju, C., Spener, F., Kofeler, H. C. & Thallinger, G. G. (2011). *Bioinformatics*, **27**, 572–577. 10.1093/bioinformatics/btq699 21169379

[bb12] Heberle, F. A., Marquardt, D., Doktorova, M., Geier, B., Standaert, R. F., Heftberger, P., Kollmitzer, B., Nickels, J. D., Dick, R. A., Feigenson, G. W., Katsaras, J., London, E. & Pabst, G. (2016). *Langmuir*, **32**, 5195–5200. 10.1021/acs.langmuir.5b04562 PMC4910133 27128636

[bb13] Hodzic, A., Rappolt, M., Amenitsch, H., Laggner, P. & Pabst, G. (2008). *Biophys. J.* **94**, 3935–3944. 10.1529/biophysj.107.123224 PMC2367187 18234811

[bb14] Hristova, K. & White, S. H. (1998). *Biophys. J.* **74**, 2419–2433. 10.1016/S0006-3495(98)77950-0 PMC1299584 9591668

[bb15] Huang, C.-H. & Mason, J. (1978). *Proc. Natl Acad. Sci. USA*, **75**, 308–310. 10.1073/pnas.75.1.308 PMC411236 272647

[bb16] King, G. I., Jacobs, R. E. & White, S. H. (1985). *Biochemistry*, **24**, 4637–4645. 10.1021/bi00338a024 4063346

[bb17] Kingsley, P. & Feigenson, G. (1979). *Chem. Phys. Lipids*, **24**, 135–147.

[bb18] Kiselev, M., Lesieur, P., Kisselev, A., Lombardo, D. & Aksenov, V. (2002). *Appl. Phys. Mater. Sci. Process.* **74**, s1654–s1656.

[bb19] Klauda, J. B., Kučerka, N., Brooks, B. R., Pastor, R. W. & Nagle, J. F. (2006). *Biophys. J.* **90**, 2796–2807. 10.1529/biophysj.105.075697 PMC1414576 16443652

[bb51] Knittelfelder, O. L., Weberhofer, B. P., Eichmann, T. O., Kohlwein, S. D. & Rechberger, G. N. (2014). *J. Chromatogr. B*, **951**, 119–128. 10.1016/j.jchromb.2014.01.011 PMC3946075 24548922

[bb20] Koenig, B. W. & Gawrisch, K. (2005). *Biochim. Biophys. Acta*, **1715**, 65–70. 10.1016/j.bbamem.2005.07.006 16109383

[bb21] Kučerka, N., Nagle, J. F., Feller, S. E. & Balgavý, P. (2004). *Phys. Rev. E*, **69**, 051903. 10.1103/PhysRevE.69.051903 15244843

[bb22] Kučerka, N., Nagle, J. F., Sachs, J. N., Feller, S. E., Pencer, J., Jackson, A. & Katsaras, J. (2008). *Biophys. J.* **95**, 2356–2367. 10.1529/biophysj.108.132662 PMC2517047 18502796

[bb23] Kučerka, N., Nieh, M.-P. & Katsaras, J. (2011). *Biochim. Biophys. Acta*, **1808**, 2761–2771. 10.1016/j.bbamem.2011.07.022 21819968

[bb24] Kučerka, N., Pencer, J., Nieh, M. P. & Katsaras, J. (2007). *Eur. Phys. J. E Soft Matter Bio. Phys.* **23**, 247–254. 10.1140/epje/i2007-10202-8 17619814

[bb25] Kučerka, N., Pencer, J., Sachs, J. N., Nagle, J. F. & Katsaras, J. (2007). *Langmuir*, **23**, 1292–1299. 10.1021/la062455t PMC2720570 17241048

[bb26] Marquardt, D., Geier, B. & Pabst, G. (2015). *Membranes*, **5**, 180–196. 10.3390/membranes5020180 PMC4496639 25955841

[bb27] Marquardt, D., Heberle, F. A., Nickels, J. D., Pabst, G. & Katsaras, J. (2015). *Soft Matter*, **11**, 9055–9072. 10.1039/c5sm01807b PMC4719199 26428538

[bb28] McIntosh, T. J., Simon, S. A., Vierling, P., Santaella, C. & Ravily, V. (1996). *Biophys. J.* **71**, 1853–1868. 10.1016/S0006-3495(96)79385-2 PMC1233653 8889161

[bb29] Murugova, T. & Balgavý, P. (2014). *Phys. Chem. Chem. Phys.* **16**, 18211–18216. 10.1039/c4cp01980f 25055002

[bb30] Nagle, J. F. & Tristram-Nagle, S. (2000). *Biochim. Biophys. Acta*, **1469**, 159–195. 10.1016/s0304-4157(00)00016-2 PMC2747654 11063882

[bb31] Pabst, G., Kučerka, N., Nieh, M.-P., Rheinstädter, M. & Katsaras, J. (2010). *Chem. Phys. Lipids*, **163**, 460–479. 10.1016/j.chemphyslip.2010.03.010 20361949

[bb32] Pabst, G., Rappolt, M., Amenitsch, H. & Laggner, P. (2000). *Phys. Rev. E*, **62**, 4000–4009. 10.1103/physreve.62.4000 11088921

[bb33] Pencer, J. & Hallett, F. (2000). *Phys. Rev. E*, **61**, 3003–3008. 10.1103/physreve.61.3003 11046629

[bb34] Pencer, J., Krueger, S., Adams, C. P. & Katsaras, J. (2006). *J. Appl. Cryst.* **39**, 293–303.

[bb52] Perly, B., Smith, I. C. P., Hughes, L., Burton, G. W. & Ingold, K. U. (1985). *Biochim. Biophys. Acta Biomembranes*, **819**, 131–135. 10.1016/0005-2736(85)90203-2 4041449

[bb35] Pernot, P. *et al.* (2013). *J. Synchrotron Rad.* **20**, 660–664. 10.1107/S0909049513010431 PMC3943554 23765312

[bb36] Petoukhov, M. V., Franke, D., Shkumatov, A. V., Tria, G., Kikhney, A. G., Gajda, M., Gorba, C., Mertens, H. D. T., Konarev, P. V. & Svergun, D. I. (2012). *J. Appl. Cryst.* **45**, 342–350. 10.1107/S0021889812007662 PMC4233345 25484842

[bb37] Petoukhov, M. V., Konarev, P. V., Kikhney, A. G. & Svergun, D. I. (2007). *J. Appl. Cryst.* **40**, s223–s228.

[bb38] Petrache, H. I., Feller, S. E. & Nagle, J. F. (1997). *Biophys. J.* **72**, 2237–2242. 10.1016/S0006-3495(97)78867-2 PMC1184418 9129826

[bb39] Price, K. & Storn, R. (1997). *Dr Dobb’s J.* **220**, 18–24.

[bb40] Price, K., Storn, R. M. & Lampinen, J. A. (2006). *Differential Evolution: a Practical Approach to Global Optimization.* Berlin, Heidelberg: Springer Science and Business Media.

[bb41] Riske, K. A., Amaral, L. Q. & Lamy-Freund, M. T. (2001). *Biochim. Biophys. Acta*, **1511**, 297–308. 10.1016/s0005-2736(01)00287-5 11286973

[bb42] Schmiedel, H., Jörchel, P., Kiselev, M. & Klose, G. (2001). *J. Phys. Chem. B*, **105**, 111–117.

[bb43] Small, D. M. (1986). *Physical Chemistry of Lipids.* New York: Plenum Press.

[bb44] Smolentsev, N., Lütgebaucks, C., Okur, H. I., de Beer, A. G. F. & Roke, S. (2016). *J. Am. Chem. Soc.* **138**, 4053–4060. 10.1021/jacs.5b11776 26938772

[bb45] Storn, R. & Price, K. (1997). *J. Glob. Opt.* **11**, 341–359.

[bb46] Verkleij, A. J., Zwaal, R. F. A., Roelofsen, B., Comfurius, P., Kastelijn, D. & van Deenen, L. L. M. (1973). *Biochim. Biophys. Acta*, **323**, 178–193. 10.1016/0005-2736(73)90143-0 4356540

[bb47] Wiener, M., Suter, R. & Nagle, J. (1989). *Biophys. J.* **55**, 315–325. 10.1016/S0006-3495(89)82807-3 PMC1330473 2713445

[bb48] Wiener, M. C. & White, S. H. (1992). *Biophys. J.* **61**, 434–447. 10.1016/S0006-3495(92)81849-0 PMC1260259 1547331

[bb49] Yuan, Y. (2000). *ICIAM 99: Proceedings of the Fourth International Congress on Industrial and Applied Mathematics, Edinburgh 5–9 July 1999*, edited by J. M. Ball & J. C. R. Hunt, pp. 271–282. Oxford: Clarenden Press.

