# Supplementary material for: Joint small-angle X-ray and neutron scattering data analysis of asymmetric lipid vesicles
Source: J Appl Crystallogr. 2017 Feb 28;50(Pt 2):419–29. doi: 10.1107/S1600576717000656 (PMC5377341; doi:10.1107/S1600576717000656)
Supplement: Supplementary file 1 [file j-50-00419-sup1.pdf]

Supplementary Material

Joint SAXS/SANS Data Analysis of Asymmetric Lipid Vesicles

Barbara Eicher, Frederick A. Heberle, Drew Marquardt, Gerald N. Rechberger, John Katsaras, Georg Pabst

1 Determination of the bilayer lipid composition via GC/MS and UPLC

The determination of the total bilayer composition has been described in detail previously by [2]. For completeness, we summarize its main features below. Lipid mixture composition can be determined by gas chromatography and mass spectrometry (GC/MS) and ultra performance liquid chromatography and mass spectrometry (UPLC-MS). For GC/MS it is necessary that there are chemical or isotopic differences between chains of the constituent lipids while for UPLC-MS a difference in lipid mass is sufficient. For GC/MS (UPLC-MS) the mole fraction χ_i of a single component can be determined directly from a set of chain (lipid) peak areas P:

$$\chi_i = \frac{P_i}{\sum_j P_j}, \tag{1}$$

where P_i represents the ith chain (lipid) peak area and the denominator is the sum over all mixture components j. This relationship is strictly valid when the chain (lipid) peak area fractions vary linearly with mixture composition. A slight deviation from linearity was found which lead to the use of a standard curves (see [2] for details). UPLC-MS data was corrected by a calibration curve of 1:1:1 mole ratio of DPPC/POPC/POPG composition. In brief, samples of 0.1-100 μg with identical mole ratios of these three components were measured, peak areas integrated and an over all measurements averaged correction factor determined depending on the detectability of the single lipids. Parameter uncertainties are estimated to be less than 5%. Lipid analysis for the isotopically asymmetric samples was conducted using GC/MS (for details see [2]) and for chemically asymmetric samples was performed by UPLC-MS. UPLC-MS measurements were conducted by using an AQUITY-UPLC system (Waters, Manchester, UK) equipped with a BEH-C18-column (2.1x150 mm, 1.7 μm) (Waters) was used for sample separation as previously described [3]. A binary gradient was applied. Solvent A consisted of water/methanol (1/1, v/v), solvent B was 2-propanol. Both solvents contained phosphoric acid (8 μM), ammonium acetate (10 mM) and formic acid (0.1 vol%). A SYNAPT^{TM}G1 qTOF HD mass spectrometer (Waters) equipped with an ESI source was used for analysis. Data acquisition was done by the MassLynx 4.1 software (Waters), for lipid analysis the 'Lipid Data Analyser' software [1] was used.

2 Evaluation of bilayer asymmetry via ^{1}H-NMR

The evaluation of the bilayer asymmetry was presented in detail in [2]. In short, ^{1}H-NMR spectra were collected on an Avance III 300 or 400 MHz spectrometer (Bruker, Billerica, MA) using the Bruker TopSpin acquisition software, and analyzed with TopSpin 3.2. The paramagnetic lanthanide ion Pr^{3+} interacts with choline protons, shifting their resonance downfield as shown in [2]. By adding Pr^{3+} to a vesicle suspension, the shift is selective for outer leaflet protiated choline, leading to a separate resolution of the protiated choline resonances from the inner and outer leaflet [5]. The integrated area R of each resonance is proportional to the number of molecules having protiated headgroups in the corresponding leaflet. The outer leaflet peak fraction is defined as:

$$f^{out} = \frac{R^{out}}{R^{in} + R^{out}}, \tag{2}$$

where the superscripts 'out' and 'in' indicate the outer and inner leaflet. When all lipids posses protiated headgroups, f^{out} directly yields the mole fraction of all bilayer lipids found in the outer leaflet:

$$X^{out} = \frac{\sum_j N_j^{out}}{\sum_j N_j} \equiv f^{out}, \tag{3}$$

where N and N^{out} denote the number of molecules in the whole bilayer and in the outer leaflet and the summation is performed over all components of the mixture. For a bilayer with an equal number of lipids in the leaflets $X^{out} = 0.5$. We assumed that $X^{out} = 0.53$ following [4] by assuming a vesicle size of 100 nm and a bilayer thickness of 50 Å. For a sample consisting of PC lipids the assay is selective for a single species provided all other components have a deuterated choline to silence their signal. If only one mixture component possesses a protiated choline we define the single-component outer leaflet peak fraction f_i^{out} as:

$$f_i^{out} = \frac{N_i^{out}}{N_i} = \frac{X^{out}\chi_i^{out}}{\chi_i}, \tag{4}$$

where χ_i^{out} stands for the outer leaflet mole fraction of component i. Combining the two previous equations gives the following expression for the outer leaflet mole fraction of component i:

$$\chi_i^{out} = \frac{f_i^{out}\chi_i}{f^{out}}. \tag{5}$$

For a two component bilayer (e.g. lipid A and B), all compositional parameters $\chi_i^{out,in}$ can be expressed as:

$$\begin{array}{lcl} \chi_A^{out} & = & \frac{f_A^{out}\chi_A}{X^{out}} \\ \chi_A^{in} & = & \frac{(1-f_A^{out})\chi_A}{(1-X^{out})}. \\ \chi_B^{in(out)} & = & 1 - \chi_A^{in(out)} \end{array} \tag{6}$$

3 Structural Data

Table S1: Structural parameters for symmetric POPC vesicles at 20 °C obtained from the slab-model. Values in parenthesis are determined by setting p_{CG} as a fit parameter. SANS data were previously published in [2] and are reanalyzed here.

	$Slab_{SAXS}$ *	$Slab_{SANS}$ †	$Slab_{Joint}$ ‡
V_{lipid} [Å^3]	1247	1247	1247
V_{head} [Å^3]	331	331	331
A_L (Å^2)	65.3 (67.6§)	67.5	67.5 (67.5§)
n_W	8.4 (8.4§)	7.9	9.9 (8.4§)
p_{CG}	1 (0.77§)	-	1 (0.55§)
p_M	0.63 (0.54§)	-	0.68 (0.46§)
χ^2_{red}	1.1 (1.1§)	94.5	54.2 (50.9§)

* Analysis of SAXS data only.
† Analysis of different contrasts of SANS data only.
‡ Joint Analysis of SANS and SAXS data.
§ p_{CG} was set as a fit parameter.

Table S2: Structural parameters of symmetric POPC vesicles 20 °C determined with the SDP-model. SANS data were previously published in [2] and are reanalyzed here.

	SAXS *	Joint Analysis$_{SANS}$ †	Joint Analysis$_{SANS\&SAXS}$ ‡
V_{lipid}§ [Å^3]	1247	1247	1247
V_{head}§ [Å^3]	331	331	331
V_{PC}§ [Å^3]	191.98	191.98	191.98
V_{CG}§ [Å^3]	139.02	139.02	139.02
V_{MN}§ [Å^3]	811.37	811.37	811.37
V_M§ [Å^3]	104.63	104.63	104.63
A_L [Å^2]	63.7	66.8	66.3
σ_{PC}	2.60	2.68	2.62
σ_{CG}	2.34	2.52	2.55
σ_M§	2.02	2.02	2.02
σ_{MN}	5.04	5.13	5.14
$\|z_{PC}\|$	19.41	18.34	18.94
$\|z_{CG}\|$	15.38	14.71	14.81
$\|z_M\|$§	1.00	1.00	1.00
$\|z_{Mn}\|$	14.38	13.71	13.81
χ^2_{red}	0.6	142.2	34.6

* Analysis of SAXS data only.
† Analysis of different contrasts of SANS data only.
‡ Joint analysis of SANS and SAXS data.
§ fixed parameter

Table S3: Leaflet composition of isotopic aLUVs determined by GC/MS and ^{1}H-NMR. χ_i indicates the total bilayer mole fraction, f_i^{out} (protiated) the fraction of a given component found in the outer leaflet and χ^{out} (χ^{in}) represents the mole fraction of all components in outer and inner leaflet. Data were previously published in [2] and are reanalyzed here.

Component	χ_i	f^{out}	χ^{out}	χ_i^{in}	χ_i^{out}
POPC^{acc}	0.64*	0.29†	0.53‡	0.96 (0.45§)	0.35 (0.19§)
POPC-d44^{don}	0.36*			0.04 (0.02§)	0.65 (0.34§)
Total	1.00			1.00 (0.47§)	1.00 (0.53§)
POPC-d44^{acc}	0.62*		0.53‡	0.83 (0.40§)	0.43 (0.23§)
POPC^{don}	0.38*	0.79†		0.17 (0.08§)	0.57 (0.30§)
Total	1.00			1.00 (0.47§)	1.00 (0.53§)

* from GC/MC
† from ^{1}H-NMR
‡ calculated for 100 nm vesicles assuming 50 Åbilayer thickness
§ total bilayer mole fraction

Table S4: Structural parameters for isotopic asymmetric POPC LUVs at 20 °C obtained from the slab model. SANS data were previously published in [2] and are reanalyzed here.

	SAXS*	Joint Analysis$_{SANS}$*	Joint Analysis$_{SANS\&SAXS}$*
V_{lipid} [Å^3]	1247	1247	1247
V_{head} [Å^3]	331	331	331
A_L^{out} [Å^2]	66.7 (65.3§)	65.4 (64.6§)	65.5 (63.8§)
A_L^{in} [Å^2]	66.7 (68.3§)	65.4 (66.6§)	65.5 (67.8§)
$n_{W_{out}}$	13 (12.6§)	4.7 (2.3§)	6.4 (4.6§)
$n_{W_{in}}$	7.0 (7.4§)	4.6 (6.2§)	6.4 (8.6§)
p_{CG}	1.0 (1.0§)	-	1.0 (1.0§)
p_M	0.67 (0.67§)	-	0.58 (0.58§)
χ^2_{red}	1.34 (1.33§)	16.3 (13.0§)	7.0 (6.3§)

* Analysis of SAXS data only (it cannot not be distsinguished between inner and outer leaflet by fit SAXS data only.)
† Analysis of different contrasts of SANS data only.
‡ Joint analysis of SANS and SAXS data.
§ Unconstrained (A_L^{in} is allowed to exceed A_L^{out} as well as $n_{w_{out}}$ can be smaller to $n_{w_{in}}$).

Table S5: Structural parameters obtained by SDP-analysis at 20 °C for isotopic aLUVs. SANS data were previously published in [2] and are reanalyzed here.

	SAXS*	Joint Analysis$_{SANS}$†	Joint Analysis$_{SANS\&SAXS}$‡
V_{lipid}§ [Å^3]	1247	1247	1247
V_{head}§ [Å^3]	331	331	331
V_{PC}§ [Å^3]	191.98	191.98	191.98
V_{CG}§ [Å^3]	139.02	139.02	139.02
V_{MN}§ [Å^3]	811.37	811.37	811.37
V_M§ [Å^3]	104.63	104.63	104.63
A_{out} [Å^2]	64.5	66.9	65.7
A_{in} [Å^2]	63.2	63.5	63.4
σ_{PCin}	2.655	2.59	2.70
σ_{PCout}	2.5413	2.68	2.57
σ_{CGin}	2.53	2.56	2.52
σ_{CGout}	2.51	2.56	2.44
σ_M§	2.02	2.02	2.02
σ_{MN}	5.14	5.08	5.04
$\lvert z_{PCin}\rvert$	18.77	20.09	19.10
$\lvert z_{PCout}\rvert$	19.58	19.14	19.87
$\lvert z_{CGin}\rvert$	15.49	15.43	15.44
$\lvert z_{CGout}\rvert$	15.20	14.69	14.94
$\lvert z_M\rvert$§	1.00	1.00	1.00
$\lvert z_{MNin}\rvert$	14.49	14.43	14.44
$\lvert z_{MNout}\rvert$	14.20	13.69	13.94
χ^2_{red}	4.5	8.7	6.6

* Analysis of SAXS data only.
† Analysis of different contrasts of SANS data only.
‡ Joint analysis of SANS and SAXS data.
§ fixed parameter

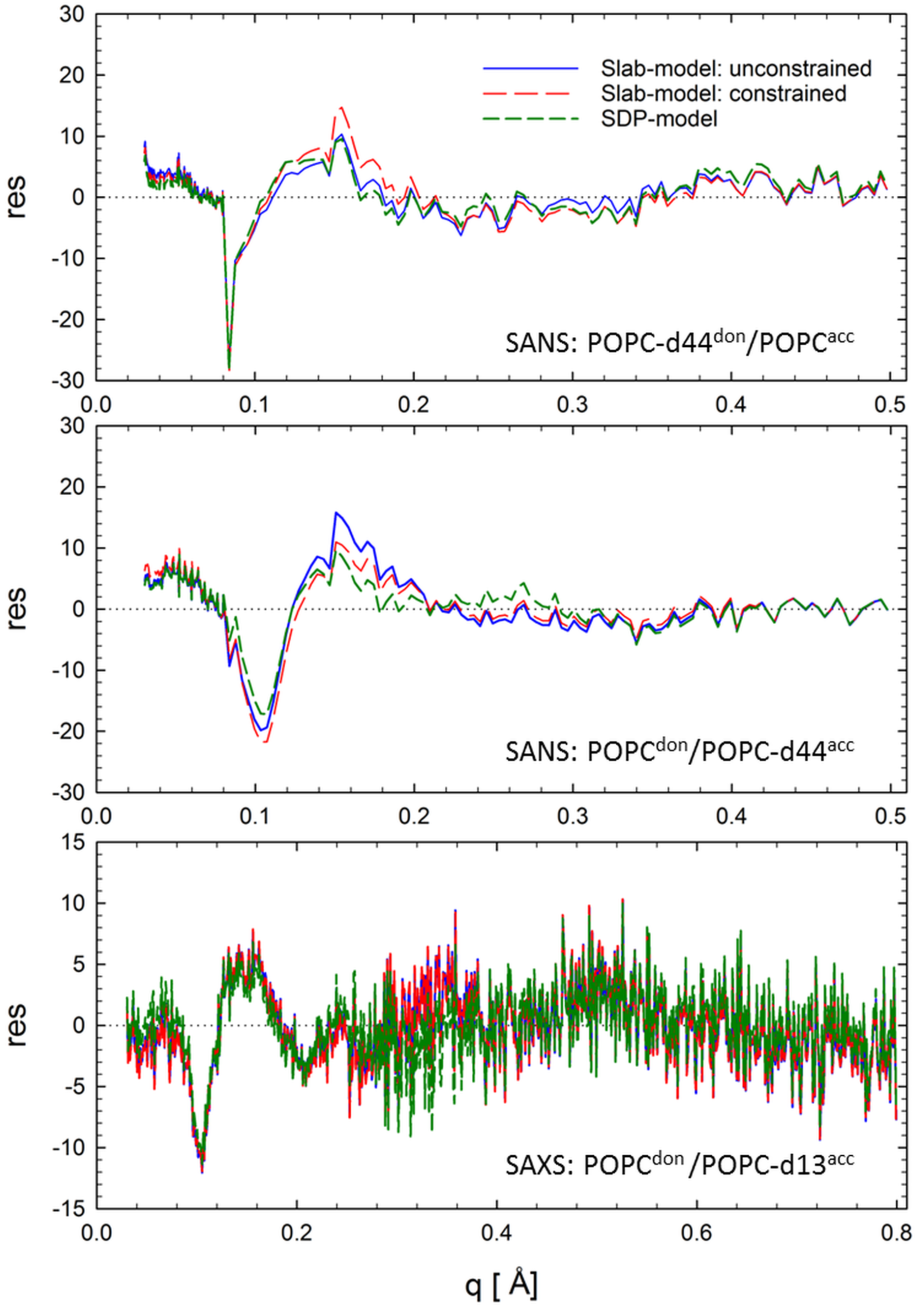

Figure S1: Residuals of isotopic aLUVs obtained by either applying the slab model without constraints (blue line), constraining the fit to $n_W^{in} < n_W^{out}$ and $A_L^{in} < A_L^{out}$ (red dashed line) or applying the SDP-model (green short-dashed line) for POPC-d44^{don}/POPC^{acc} (top panel), POPC^{don}/POPC-d44$_{acc}$ (middle panel) and for SAXS experiments (lower panel). It can be seen that the difference in residuals of the constrained and unconstrained fits cannot be distinguished. SANS data were previously published in [2] and are reanalyzed here.

Table S6: Leaflet composition of chemical aLUVs determined by UPLC-MS and ^{1}H-NMR. χ_i^{out} (χ_i^{in}) indicates the mole fraction of all components in outer and inner leaflet.

	Component	χ_i	χ^{out}	χ_i^{in}	χ_i^{out}
	POPC-d13 (acceptor)	0.72*	0.53‡	0.92 (0.43§)	0.57 (0.29§)
SANS	DPPC-d64 (donor)	0.28*		0.08 (0.04)	0.43 (0.24)
	Total	1.00		1.00	1.00
	POPC-d13 (acceptor)	0.74*	0.53‡	0.85 (0.40§)	0.65 (0.34§)
SANS	DPPC (donor)	0.26*		0.15 (0.07§)	0.35 (0.19§)
	Total	1.00		1.00	1.00
	POPC-d13 (acceptor)	0.73*	0.53‡	0.91 (0.43§)	0.57 (0.30§)
SAXS	DPPC (donor)	0.27*		0.09 (0.04§)	0.43 (0.23§)
	Total	1.00		1.00	1.00

* from UPLC
‡ calculated for 100 nm vesicles assuming 50 $\AA$ bilayer thickness
§ total bilayer mole fraction

Table S7: Structural parameters of chemical DPPC/POPC aLUVs obtained by the slab model at 50 °C.

	SAXS*		Joint Analysis$_{SANS}$†		Joint Analysis$_{SANS\&SAXS}$‡	
	DPPC	POPC	DPPC	POPC	DPPC	POPC
V_{lipid}§ [Å^3]	1228.5	1275.5	1228.5	1275.5	1228.5	1275.5
V_{head}§ [Å^3]	331	331	331	331	331	331
A_L [Å^2]	64.9	69.7	61.7	65.7	61.7	68.7
n_W	6.6	6.6	7.2	7.2	4.7	4.3
p_{CG}§	1.00		-		1.00	
p_M	0.54		-		0.49	
χ^2_{red}	31.1		323.5		137.8	

* Analysis of SAXS data only.
† Analysis of different contrasts of SANS data only.
‡ Joint analysis of SANS and SAXS data.
§ fixed parameter

Table S8: Structural parameter of scrambled DPPC/POPC LUVs determined by the slab model at 50 °C.

	SAXS*		Joint Analysis$_{SANS}$†		Joint Analysis$_{SANS\&SAXS}$‡	
	DPPC	POPC	DPPC	POPC	DPPC	POPC
V_{lipid}§ [Å^3]	1228.5	1275.5	1228.5	1275.5	1228.5	1275.5
V_{head}§ [Å^3]	331	331	331	331	331	331
A_L [Å^2]	63.4	68.4	64.9	66.5	64.9	67.8
n_W	5.3	4.9	3.2	3.2	5.0	5.0
p_{CG}§	1.00		-		1.00	
p_M	0.51		-		0.51	
χ^2_{red}	13.0		241.1		45.1	

* Analysis of SAXS data only.
† Analysis of different contrasts of SANS data only.
‡ Joint analysis of SANS and SAXS data.
§ fixed parameter

Table S9: Structural parameters of chemical aLUVs determined by the SDP model at 50 °C.

	SAXS*		Joint Analysis$_{SANS}$†		Joint Analysis$_{SANS\&SAXS}$‡	
	DPPC	POPC	DPPC	POPC	DPPC	POPC
V_{lipid}§ [Å^3]	1228.5	1275.5	1228.5	1275.5	1228.5	1275.5
V_{head}§ [Å^3]	331	331	331	331	331	331
V_{PC}§ [Å^3]	191.98	191.98	191.98	191.98	191.98	191.98
V_{CG}§ [Å^3]	139.02	139.02	139.02	139.02	139.02	139.02
V_{MN}§ [Å^3]	787.77	835.54	787.77	835.54	787.77	835.54
V_M§ [Å^3]	109.73	108.54	109.73	108.54	109.73	108.54
A_L [Å^2]	62.2	64.9	62.2	66.6	62.6	67.9
σ_{PC}	2.37	4.37	2.37	2.65	2.34	2.61
σ_{CG}	2.18	2.38	2.21	2.50	2.09	2.55
σ_M§	2.38	2.02	2.38	2.02	2.38	2.02
σ_{MN}	5.68	5.20	5.54	5.13	5.43	5.13
$\lvert z_{PC}\rvert$	19.18	16.64	19.97	19.41	19.71	17.12
$\lvert z_{CG}\rvert$	15.42	15.56	15.42	15.16	15.33	14.91
$\lvert z_M\rvert$§	1.00	1.00	1.00	1.00	1.00	1.00
$\lvert z_{MN}\rvert$	14.42	14.56	14.42	14.16	14.33	13.91
χ^2_{red}	4.2		303.1		141.3	

* Analysis of SAXS data only.
† Analysis of different contrasts of SANS data only.
‡ Joint analysis of SANS and SAXS data.
§ fixed parameter

Table S10: Structural parameters of scrambled DPPC/POPC LUVs determined by the SDP model at 50 °C.

	SAXS*		Joint Analysis$_{SANS}$†		Joint Analysis$_{SANS\&SAXS}$‡	
	DPPC	POPC	DPPC	POPC	DPPC	POPC
V_{lipid}§ [Å^3]	1228.5	1275.5	1228.5	1275.5	1228.5	1275.5
V_{head}§ [Å^3]	331	331	331	331	331	331
V_{PC}§ [Å^3]	191.98	191.98	191.98	191.98	191.98	191.98
V_{CG}§ [Å^3]	139.02	139.02	139.02	139.02	139.02	139.02
V_{MN}§ [Å^3]	787.77	835.54	787.77	835.54	787.77	835.54
V_M§ [Å^3]	109.73	108.54	109.73	108.54	109.73	108.54
A_L [Å^2]	63.3	69.3	63.4	68.2	64.4	69.1
σ_{PC}PC	2.26	3.01	2.26	2.67	2.40	2.70
σ_{CG}	2.25	2.63	2.21	2.52	2.22	2.54
σ_M§	2.38	2.02	2.38	2.02	2.38	2.02
σ_{MN}	5.47	5.08	5.72	6.38	5.56	5.16
$\|z_{PC}\|$	19.62	17.79	20.24	19.41	19.32	17.64
$\|z_{CG}\|$	15.19	14.60	15.20	14.90	14.94	14.64
$\|z_M\|$§	1.00	1.00	1.00	1.00	1.00	1.00
$\|z_{MN}\|$	14.19	13.60	14.20	13.90	13.94	13.67
χ^2_{red}		1.09		61.4		18.7

* Analysis of SAXS data only.
† Analysis of different contrasts of SANS data only.
‡ Joint analysis of SANS and SAXS data.
§ fixed parameter

Table S11: Structural parameter of chemical aLUVs and scrambled LUVs for the outer/inner leaflet determined from values obtained in table S7,S8,S9, S10.

Model	Components	d_{Co} [Å]	d_{Ci} [Å]	d_B [Å]	A_L^{out} [Å^2]	A_L^{in} [Å^2]
	$SANS_1$*	14.11	13.81	37.74	66.5	68.1
$AsymSlab_{Joint}$	$SANS_2$†	14.03	13.87	37.79	66.2	67.6
	$SAXS_1$‡	14.09	13.82	37.82	65.6	68.1
	$SANS_1$*	14.09	14.01	38.09	65.5	67.1
$AsymSDP_{Joint}$	$SANS_2$†	14.05	13.97	37.96	66.0	67.1
	$SAXS_1$‡	14.09	13.94	37.98	65.6	67.4
	$SANS_1$*	13.90	13.90	37.68	67.0	67.0
$ScramSlab_{Joint}$	$SANS_2$†	13.90	13.90	37.68	67.1	67.1
	$SAXS_1$‡	13.90	13.90	37.68	67.1	67.1
	$SANS_1$*	13.91	13.91	37.26	67.8	67.8
$ScramSDP_{Joint}$	$SANS_2$†	13.91	13.91	37.22	67.9	67.9
	$SAXS_1$‡	13.90	13.90	37.25	67.8	67.8

* DPPC-d64^{don}/POPC-d13^{acc} (SANS)
† DPPC^{don}/POPC-d13^{acc} (SANS)
‡ DPPC^{don}/POPC-d13^{acc} (SAXS)

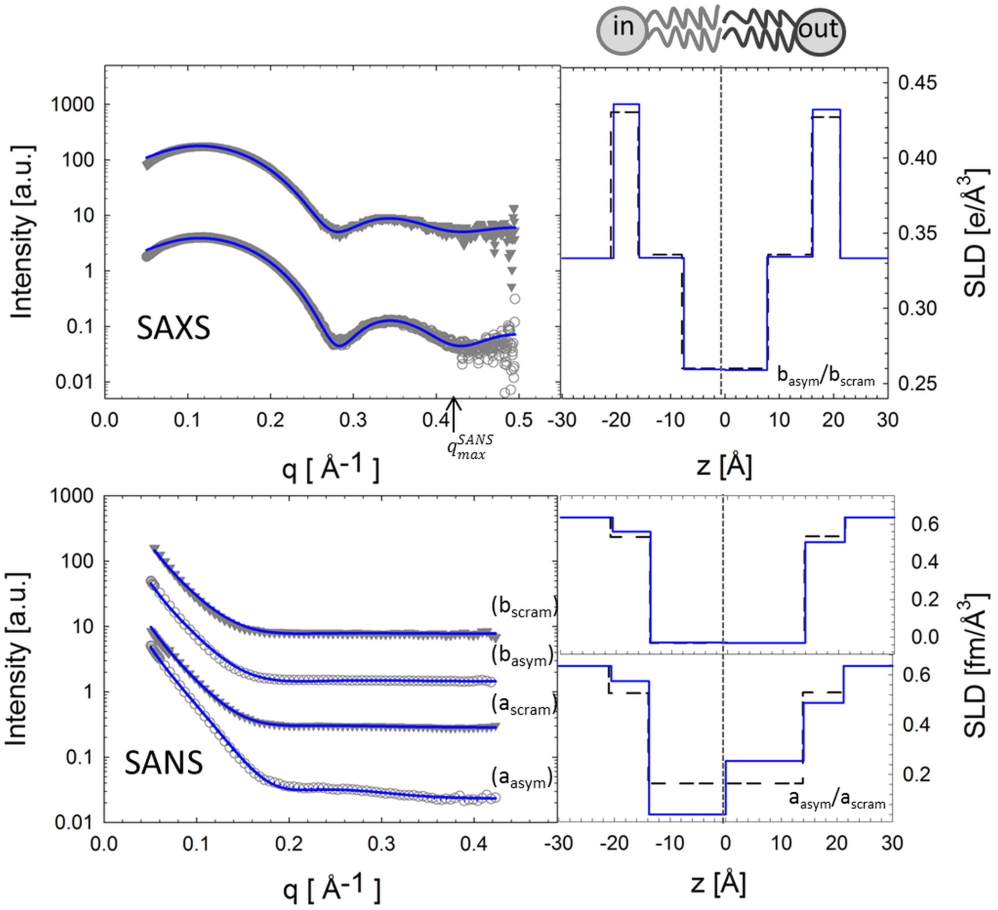

Figure S2: Slab analysis (blue lines) of DPPC^{don}/POPC^{acc} aLUVs (open circles) and scrambled LUVs (filled triangles). Panels on the right show the corresponding SLDs (blue: aLUVs; dashed: scrambled LUVs). The different contrast samples for SANS experiments were DPPC-d62^{don}/POPC-d13^{acc} (a_{asym}/a_{scram}) and DPPC^{don}/POPC-d13^{acc} (b_{asym}/b_{scram}). Data were offset vertically for clarity.

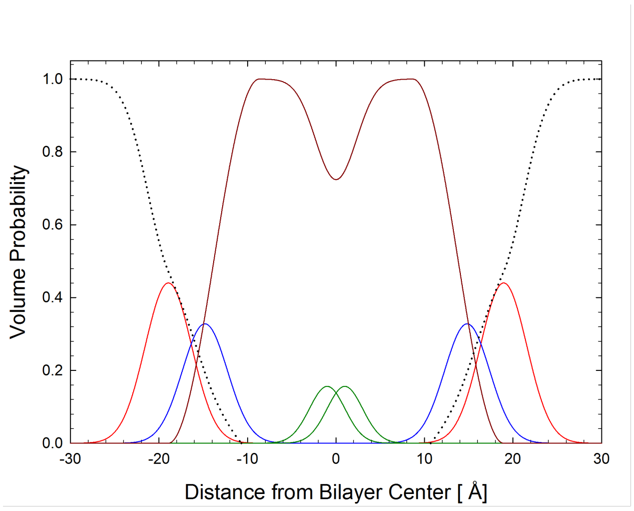

Figure S3: SDP analysis of POPC-LUVs measured at 20 °C, showing the volume probability distribution of the joint ananylsis of SANS and SAXS data.

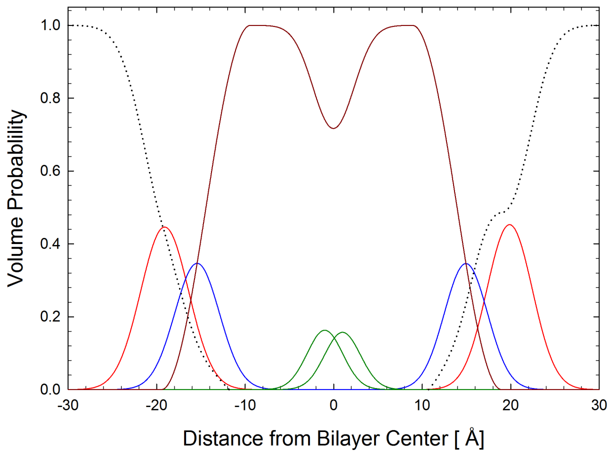

Figure S4: SDP analysis of POPC-aLUVs measured at 20 °C, showing the volume probability distribution of the joint ananylsis of SANS and SAXS data.

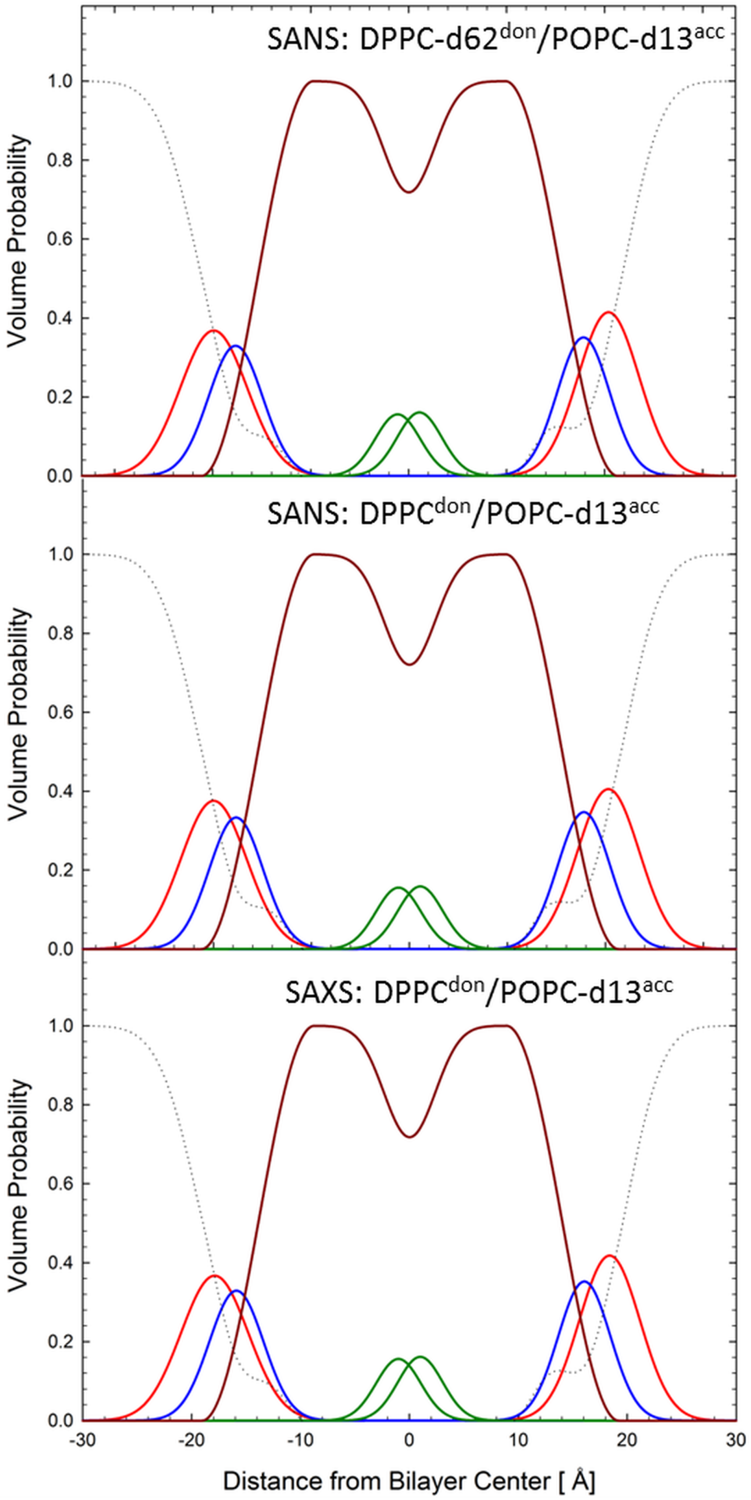

Figure S5: SDP analysis of chemical-aLUVs measured at 50 ℃, showing the volume probability distribution of the joint analysis of the structure of DPPC/POPC for different conrasts (upper two panels for SANS measurements, lower panel SAXS) which show a slightly different exchange efficiency.

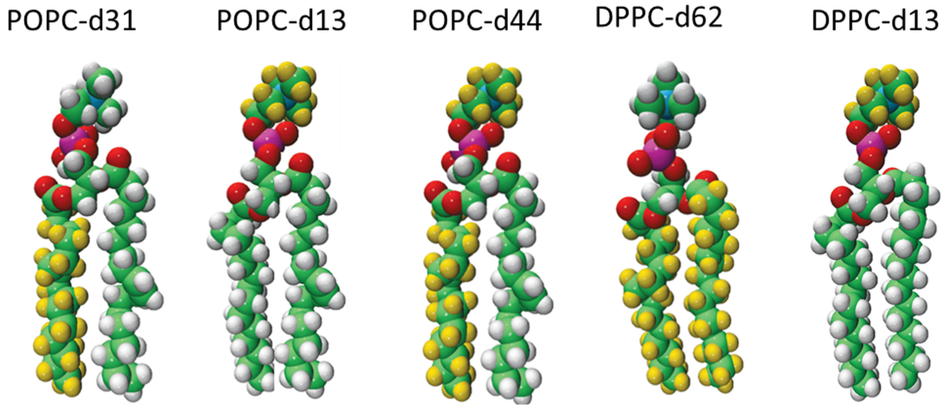

Figure S6: Chemical structures of deuterated phospholipids. Lipids are displayed in space fill representation: white indicating hydrogen and yellow deuterium.

References

[1] Jürgen Hartler, Martin Trötzmüller, Chandramohan Chitraju, Friedrich Spener, Harald C Köfeler, and Gerhard G Thallinger. Lipid data analyzer: unattended identification and quantitation of lipids in lc-ms data. *Bioinformatics*, 27(4):572–577, 2011.

[2] Frederick A Heberle, Drew Marquardt, Milka Doktorova, Barbara Geier, Robert F Standaert, Peter Heftberger, Benjamin Kollmitzer, Jonathan D Nickels, Robert A Dick, Gerald W Feigenson, et al. Sub-nanometer structure of an asymmetric model membrane: Interleaflet coupling influences domain properties. *Langmuir*, 32(20):5195–5200, 2016.

[3] Oskar L Knittelfelder, Bernd P Weberhofer, Thomas O Eichmann, Sepp D Kohlwein, and Gerald N Rechberger. A versatile ultra-high performance lc-ms method for lipid profiling. *Journal of Chromatography B*, 951:119–128, 2014.

[4] Drew Marquardt, Barbara Geier, and Georg Pabst. Asymmetric lipid membranes: Towards more realistic model systems. *Membranes*, 5(2):180–196, 2015.

[5] B. Perly, I. C. P. Smith, L. Hughes, G. W. Burton, and K. U. Ingold. Estimation of the location of natural α-tocopherol in lipid bilayers by 13c-nmr spectroscopy. *Biochimica et Biophysica Acta (BBA) - Biomembranes*, 819(1):131–135, 9/25 1985.
